# Some Peculiarities of Doctors

**Published:** 1898-11

**Authors:** 


### SOME PECULIARITIES OF DOCTORS.

Doctors are in some respects very incomprehensible people. They are over-sensitive, and prone to misunderstand the motives of others. They do and say things which other people would not do or say. Many things which others think proper they think improper. This is especially true in business matters. The training of a physician is very different from that of an ordinary business man. He spends years in college and a professional school, and when he gets into active practice he still lives in a world of thought, while the business man lives in a world of practical activity. Two men who were bosom friends as boys and who have become, one a physician and the other a business man, find when they are forty that they are in many ways strangers to each other. Their time has been spent in such different ways that they have grown apart, and in many things do not understand each other. While the one in his quiet study was acquainting himself with the ideas and actions of men who have been dead perhaps for centuries, the world has, so to speak, passed along with a clatter and strife which he quite likely comes to despise. His companion, on the other hand, has entered the rushing throng and struggles to keep his place in its onward march. One becomes a man of thought—the other a man of action. Just in proportion as each has devoted himself exclusively to his own line of work, he becomes incapable of sympathizing with his friend or even understanding him. The doctor seems to his friend to become "funny," then "rather queer," and at last "decidedly foolish;" while the business man who was perhaps in boyhood the soul of integrity, seems to the doctor, who does not understand modern methods of business or even the fundamental principles on which alone a successful career can be built, to have lost much of the sense of honor. So they continue to grow apart except as the requirements of business or social intercourse bring them together and enable them to see why the other thinks and acts as he does.

Too often the doctor's intercourse with business men is too limited to enable him ever to understand them, and he continues through life to hold his "queer" ideas of business. If he has on hand a matter of business so simple that a common office boy would know just what to do, he feels obliged often to seek advice before undertaking it, but too frequently he thinks this would be humiliating or assumes that he knows enough to get on without advice, or that men in doing business with him will think of his advantage and profit as well as their own. Usually this ends in trouble or loss of some kind and sometimes in annoying lawsuits. A doctor is not as likely to be aroused by such an experience to acquaint himself with business methods as he is to become embittered against business and business men, and to think they all lack honor and honesty.

Doctors also show how different they are from other men in that the plain statement of these facts will often call forth keen resentment from many of them. Possibly that will be the effect of this editorial. Lawyers will oppose each other in court with all their might and apparently with great bitterness, and yet in a few minutes be seen on the street chatting together on the best of terms. Clergymen are like doctors in this respect, and with them form one extreme as lawyers form the other. The difference between them is this: Lawyers distinguish between *personality* and *opinion;* clergymen and doctors do not, as a rule. The lawyers are right. You doubtless have friends who hold views radically different from yours in some respects and yet you remain firm friends. If you ever criticise each other you do it without any idea that the other is unworthy of your respect and friendship. You distinguish in that one instance between *personality* and *opinion.* You differ with your friend's *opinion* and criticise it, but respect his *personality.* You see that it is possible for him to be wrong in his *opinion,* while remaining noble and true in character.

Every one has friends whom he treats in this way, but the fact is, the number of subjects on which clergymen and doctors will allow others to differ with them, while still counting them friends and retaining respect for them, is comparatively small. With business men the number of such subjects is much larger, and with lawyers greater than any other class. The matter resolves itself into the degree of *tolerance* for the opinion of others. The clergy was the most intolerant class in the community in the middle ages, as the tortures of the inquisitions and burnings at the stake testify. Probably they hold that place to-day, but doctors as a class are nearer to them than they are to business men or lawyers. The training which each class receives determines its characteristics. It is not a question of men, but of the effect of training and environment long continued, as pointed out above. Very likely your studies have made you a firm believer in the doctrine of evolution, that "function determines development;" if so, you will readily see why these classes differ in this way. The clergyman studies *absolute right,* and tries to measure all human action by that standard, and becomes an uncompromising foe of everything that differs with it. He may meet a good many men, but he sees as a rule only their Sunday clothes and their Sunday thoughts; he sees the best side of their natures. Lawyers, on the other hand, see the worst side of men; they become acquainted with the different ideas of right that prevail in different nations and even among different classes in the same community, and find that men, noble men, honestly hold opinions which are directly antagonistic, and so lawyers come quite naturally to distinguish between persons and opinions more fully than any other class.

It is because we firmly believe that doctors can command the respect of communities in which they live, especially of business men, to a far greater extent than they do at present, that we speak thus strongly. Doctors, as a rule, are exceedingly good fellows, and we would be the last to point out their shortcomings, except in a friendly spirit. They occupy positions of prominence in their communities, and so every little foible which they may possess will be the more conspicuous.

Perhaps their peculiarities are most noticeable in business matters. As a rule their business transactions are limited to collecting bills and buying the supplies which their families require. In the latter they are often as successful as other men, because their experience usually begins as early in life. But in collecting doctors sometimes exhibit an amazing ignorance of human nature. Young graduates often think all they have to do to get ahead in life is to attend a few "cases," have some bills printed from a steel plate on nice paper, and when they want money fill one of the blanks and send it to a patient and wait for the next return mail to bring them a check. This delusion does not last long. Other physicians, whose years of experience should have taught them wisdom, never send bills till they need the money. If they happen to want one hundred dollars they will look over their books and find some one who owes them about that and send a bill. They never send bills to patients who are "good" until they actually need money, and then are surprised to find some have moved away, some forgotten that they were ever treated at all, and others inclined to claim that the bill was paid "long ago," because it is so old and "gray-haired," and it is not their custom to allow bills to remain unpaid so long. As a result the doctor finds that he is lucky if he can collect 25 per cent. or perhaps even 10 per cent. of the amount on his ledger. To some extent the public is to blame for this, but it will do little good to discuss that point here, as few laymen will see this article. Doctors could themselves, however, obtain very much better results if they would study the methods of collecting. It is not a difficult question to master. For a doctor to be as ignorant of collecting as his collector is of obstetrics is a disgrace. No matter how careful a surgeon is to boil his instruments, to use only distilled water and have everything about the operating room antiseptic, if he forgets to wash his hands or leaves any one of the steps even "half done" his work is not perfect. In the same way it may be said that however successful as a practitioner a doctor may be, if he fails to collect enough from his practice to pay his honorable debts he is nevertheless something of a failure as a *man* and *citizen.* Doctors deal with their fellow-men at two points—the sick bed and the cashier's desk. If they are "slow" at the latter point people will say it is because they are "slow" in the other; whereas the fact is more likely to be that they believe in the Golden Rule themselves and think their patients are living in accordance with its precept, and will be only too glad to pay their doctor's bill the moment he gives them a chance. Such faith in the good intentions of humanity would be sublime if it were only well-founded. But it does not require a long experience in business to discover that while most men consider the highest rule of life to be "Do unto others as ye would that they should do unto you," they are living in accordance with the vulgar revision of that rule, "Do others before they do you." We do not mean by this that most men are dishonest, but simply that the customs of business have not as yet been evolved as far from primeval crudeness as our ethical precepts. Office boys learn business customs through the week and ethical principles on Sunday, and when in later years they are looked up to through the week as "very successful" and on Sunday as "very pious," they may not be conscious of this great discrepancy between their practices and their professions.

Some doctors work for fame; they want to be known in their localities as the most successful surgeons, or general practitioners, or specialists in some particular line; but most doctors are practicing to make a living. It would be well, therefore, if they could bring themselves to feel that no case should be considered as "successfully treated" until they have collected the bill or remitted it for charity's sake. To them collecting is a very vital point of practicing. It would be well if medical schools gave a few lectures on this subject and spared young doctors their costly experience.

Business men usually send a bill with goods when they are delivered or soon after, and a statement of account on the first of the following month and each month thereafter until the bill is paid. They expect to receive bills for what they owe in the same way. It is romantic nonsense for doctors to suppose that intelligent people will be offended if they send a bill within a year. You will no doubt find exceptions, but can you afford to sacrifice one-quarter or one-half of what you can collect by acting promptly like a business man, for the sake of the good will of such unreasonable persons? The doctor who arrives at a general conclusion that because exceptional individuals are offended by bills within a year, all his patients will be, is very illogical. His mind would not act so in professional matters. He would not say that because one man swallowed a fish-bone "without hearing from it," all men could do the same. You can not escape the criticism of cranks, if you try. Wait a year and some will forget how serious their illness was and so will refuse to settle unless the bill is reduced; send your bill while the patient is still pale and keenly conscious of the seriousness of his sickness and you will be criticised for not giving for him more time to "get on his feet again" in business. You must steer between Scylla and Charybdis. Many doctors think that by sending bills soon after patients recover they will give offense and cause them to select another physician the next time they are ill. But is it not equally true that if you wait long before sending bills you will find that some men by reverses of fortune or lack of thrift have become unable to pay their bills and are so ashamed of themselves for allowing them to remain unpaid that they will seek medical advice elsewhere? It is better to lose the former patient than the latter. We once knew a doctor who never allowed an account to remain long unsettled. If he found a patient too poor to pay a remunerative fee he scaled his bill down to meet the patient's ability. Sometimes he donated his services, but he saw to it that in some way every bill was settled. The consequence was that while he had the reputation of being a close financier, he drove the best and fastest horses, had the best carriages, lived in a large house with fine grounds and had more friends than any other man in the community. Every one knew that he could command the doctor's services and also that he would have to pay him according to his ability—a good fee if he was well off and nothing at all if he were very poor. That doctor left a snug fortune when he died. It would be well for the profession if more doctors would follow his example. Perhaps it would prove a good thing for the doctors in each community to combine and adopt a uniform system of collecting and barring the chronic deadbeats from getting any treatment until they deal fairly with physicians. Certainly it would be a good thing for the reputations, practice and bank accounts of the doctors themselves if they could succeed in conducting their business affairs more nearly as business men do.

We speak strongly on this subject because we think it is of vital importance in the success of every practitioner. We should be very glad to publish the views of any doctors who differ with us or the experiences of any who have discovered successful ways of collecting. If requested we would withhold the names of correspondents.

